# Mesothelioma diagnosis-still a challenge

**DOI:** 10.36416/1806-3756/e20240118

**Published:** 2024-05-08

**Authors:** Ubiratan de Paula Santos

**Affiliations:** 1. Divisao de Pneumologia, Instituto do Coracao - InCor - Hospital das Clínicas, Faculdade de Medicina, Universidade de Sao Paulo, Sao Paulo (SP) Brasil.

Even though malignant mesothelioma (MM) was first identified and named in 1931,^1^ and its link to asbestos exposure has been established since 1960,^1^ ^,^ ^2^ MM diagnosis and, as a result, its registry closer to reality and its treatment are still a challenge. In this current issue of the *Jornal Brasileiro de Pneumologia*, an interesting paper discusses the obstacles that hospitals in the State of São Paulo, Brazil, face in identifying MM and suggests recommendations to reduce uncertainty or diagnostic error.^3^

Although the objective of that study, as described by the authors,^3^ was to create a pathology board of experts and review the diagnosis of possible cases and/or occult cases of MM retrieved from the Hospital-Based Cancer Registry database in the State of São Paulo, it also addresses the diagnostic accuracy of MM seen in the hospitals of that State. Their motivation seeks an answer to the low number of mesothelioma diagnoses considering the amount of asbestos that is consumed in Brazil, one of the highest worldwide. From the 1970s to the early 2000s, asbestos consumption was above 1 kg *per capita*,^4^ and it is estimated that Brazil consumed around six million tons of asbestos between 1961 and 2012.^5^ The low number of MM cases that are diagnosed stands in contrast to the findings of several studies that reveal an association between the amount of asbestos consumed in countries or regions and the incidence of mesothelioma.^5^ ^,^ ^6^

Underreporting may result from lack of assessment of individuals exposed to asbestos, failure to address the history of occupational or environmental exposure, limitations in imaging analyses, insufficient biopsy material, and difficulties in pathological diagnosis. It is recognized that the histopathological diagnosis of mesothelioma is not straightforward: it is usually complex, requiring the combination of experienced pathologist/pathology service, and, in order to confirm more difficult cases, it is always appropriate for a chest radiologist, an oncologist, and a pulmonologist with expertise in occupational respiratory diseases to be part of the medical staff. Nonetheless, this reality is uncommon in the services that treat, diagnose, and register the majority of cases.

That study, unprecedented in Brazil,^3^ revealed the need to review 27% of cases (130 out of 482), which presented topography and/or morphology aspects that were compatible with MM, but with insufficient pathological criteria for diagnosis. Of those 130, 73 biopsy specimens that had topography and/or morphology compatible with MM, but lacked sufficient pathological criteria for a diagnosis, were made available, from 11 of the 25 solicited hospitals, for the expert panel examination. The analyses confirmed 9 cases with MM (12.3% of the 73 cases reviewed), 58 cases (79.5%) had an MM diagnosis excluded, 2 of which had previously been established as MM, and others (n = 6; 8.2%) were found to be inconclusive. In addition to the complexity of the topic since the diagnosis of mesothelioma, in many situations, does not allow for certainty, the study revealed important limitations that make the diagnosis and registration of mesothelioma in Brazil flawed.

That study^3^ showed that, in some of the hospitals evaluated, there is a lack of pathology services with expertise in the subject, in addition to presenting evidence of inadequate procedures for the storage of biopsy materials, both in terms of time, which, in accordance with the standardization in the State of São Paulo, should be stored for 5 years, and in terms of quantity and quality of materials to allow adequate reanalysis. It is remarkable that 14 of the 25 hospitals selected to provide materials for the study neither cooperated nor provided any materials.

That article^3^ highlights the need to establish regulations for hospitals that care for patients where the diagnostic hypothesis of mesothelioma is imposed, in addition to the use of appropriate histopathological criteria for diagnosis, which requires adequate tissue sampling and the use of a panel with immunohistochemistry markers.^7^ ^,^ ^8^ The relatively high number of inconclusive cases (8%) in the sample evaluated suggests the need to persist in refining biomarkers in order to improve the accuracy of MM diagnosis, as well as the creation of a panel of experts comprising pathologists, pulmonologists with experience in occupational and environmental areas, oncologists, and radiologists to confirm the diagnosis of more complex cases. The importance of an accurate and timely diagnosis impacts the treatment that affects patient survival, the possibility for the patient to claim (or not) their rights with public insurance bodies, and the advancement of understanding the epidemiology of mesothelioma in Brazil.^9^ ^,^ ^10^
